# NADP-Dependent Aldehyde Dehydrogenase from Archaeon* Pyrobaculum sp.1860*: Structural and Functional Features

**DOI:** 10.1155/2016/9127857

**Published:** 2016-11-10

**Authors:** Ekaterina Yu. Bezsudnova, Tatiana E. Petrova, Natalia V. Artemova, Konstantin M. Boyko, Ivan G. Shabalin, Tatiana V. Rakitina, Konstantin M. Polyakov, Vladimir O. Popov

**Affiliations:** ^1^A.N. Bach Institute of Biochemistry, Research Center of Biotechnology of the Russian Academy of Sciences, Leninsky Ave. 33, Bld. 2, Moscow 119071, Russia; ^2^Institute of Mathematical Problems of Biology RAS, The Branch of Keldysh Institute of Applied Mathematics of the Russian Academy of Sciences, Pushchino, Russia; ^3^NBICS Center, National Research Centre “Kurchatov Institute”, Akad. Kurchatova Sqr. 1, Moscow 123182, Russia; ^4^Engelhardt Institute of Molecular Biology of the Russian Academy of Sciences, Vavilov Str. 32, Moscow 119991, Russia

## Abstract

We present the functional and structural characterization of the first archaeal thermostable NADP-dependent aldehyde dehydrogenase AlDHPyr1147.* In vitro*, AlDHPyr1147 catalyzes the irreversible oxidation of short aliphatic aldehydes at 60–85°С, and the affinity of AlDHPyr1147 to the NADP+ at 60°С is comparable to that for mesophilic analogues at 25°С. We determined the structures of the apo form of AlDHPyr1147 (3.04 Å resolution), three binary complexes with the coenzyme (1.90, 2.06, and 2.19 Å), and the ternary complex with the coenzyme and isobutyraldehyde as a substrate (2.66 Å). The nicotinamide moiety of the coenzyme is disordered in two binary complexes, while it is ordered in the ternary complex, as well as in the binary complex obtained after additional soaking with the substrate. AlDHPyr1147 structures demonstrate the strengthening of the dimeric contact (as compared with the analogues) and the concerted conformational flexibility of catalytic Cys287 and Glu253, as well as Leu254 and the nicotinamide moiety of the coenzyme. A comparison of the active sites of AlDHPyr1147 and dehydrogenases characterized earlier suggests that proton relay systems, which were previously proposed for dehydrogenases of this family, are blocked in AlDHPyr1147, and the proton release in the latter can occur through the substrate channel.

## 1. Introduction

Nonphosphorylating hydrolytic aldehyde dehydrogenases (AlDHs) catalyze irreversible NAD(P)+-dependent oxidation of aldehydes to carboxylic acids involving a water molecule in the deacylation step. Steady state initial velocity studies showed that the AlDH-catalyzed dehydrogenation of aldehydes follows an ordered sequential mechanism (bi-bi) [1, 2]. The kinetic analysis and structural investigation of AlDHs and their mutant forms demonstrated that the enzymatic oxidation of aldehydes involves three steps: the acylation of catalytic cysteine to thiohemiacetal, the transfer of a hydride ion to the coenzyme giving thioester, and the deacylation of the resulting thioester to form carboxylic acid [3–5]. The active site of AlDHs is formed by the catalytic Cys and Glu residues and several invariant residues responsible for the coenzyme binding and stabilization of intermediates [6–9]. There is good consensus on the role of cysteine. Thus, the thiolate ion attacks aldehyde to form thiohemiacetal intermediate followed by formation of a thioacylenzyme intermediate, and the mutations at this particular cysteine residue lead to inactivation of the enzyme [7, 10, 11]. The role of the catalytic glutamate remains unclear. It was shown that the replacement of glutamate by alanine leads to a dramatic decrease in the deacylation rate and an increase in the affinity for NADP+ [10, 12]. It is commonly accepted that Glu acts as a general base and activates a water molecule in the deacylation step [5, 12–14]. The general base catalysis by glutamate resulting in activation of Cys in the acylation step remains controversial [12, 15].

An analysis of the crystal structures of dehydrogenases revealed conformational flexibility of the catalytic residues and the nicotinamide moiety of the coenzyme [5, 6, 16]. Different steps of the reaction are characterized by different combinations of the conformation of Cys, Glu, and the coenzyme. The coenzyme is tightly held at its position through the adenine moiety, the pyrophosphate group, and both ribose units, whereas the nicotinamide moiety is fixed more weakly and displays conformational flexibility [5, 16]. The fully ordered coenzyme was obtained in complexes with inactive mutant forms. In the crystal structures of NAD(P)-dependent aldehyde dehydrogenases, two stable conformations of the coenzyme are distinguished: “hydride transfer” (PDB entry 1BPW [17] and PDB entry 1EZ0 [9]) and “hydrolysis” (PDB entry 3JZ4 [18] and PDB entry 1O02 [16]). It is believed that the “hydride transfer” (extended) conformation of NAD(P) corresponds to the acylation and the transfer of a hydride ion, whereas the “hydrolysis” (contracted) conformation is related to the deacylation step [8–10, 16]. In addition, in some structures, such as lactaldehyde dehydrogenase from* Escherichia coli* (PDB entry 2ILU [19]), glyceraldehyde-3-phosphate dehydrogenase from* Streptococcus mutans* (PDB entry 2ESD [11]), and aldehyde dehydrogenase from* Burkholderia xenovorans* (PDB entry 2VRO [20]), the coenzyme is in the “out” conformation, in which the nicotinamide moiety is outside the coenzyme entrance. Di Costanzo and coworkers suggested that the “out” conformation is either a product release conformation or an initial binding conformation [19]. In the crystal structures, the catalytic Cys and Glu residues are in different orientations in concert with the conformations of the coenzyme. Thus, Cys adopts two stable conformations, which differ in the orientation of the thiol group that is directed either inward or outward from the coenzyme-binding pocket [5, 8]. The catalytic Glu was found to adopt three stable conformations: “inside,” “intermediate,” and “outside.” In the “inside” conformation, the carboxyl group is directed inward in the catalytic site and forms a hydrogen bond with the catalytic cysteine, whereas in the other two conformations the carboxyl group is directed away from this site and forms hydrogen bonds with other invariant residues [5].

To the best of our knowledge, the inhibition of the binding of the reactive oxidized form NAD(P) by NAD(P)H was observed only for betaine aldehyde dehydrogenase from* Pseudomonas aeruginosa* (PaBADH) [21, 22]. The mechanism, by which the enzyme differentiates the oxidation states of the coenzyme, is not fully understood. This mechanism was studied in detail by Tsybovsky and coworkers for 10-formyltetrahydrofolate dehydrogenase from* Rattus norvegicus* (C_t_-FDH) [6, 23]. The authors showed that there is a transient covalent bond between the sulfur atom of the catalytic cysteine and the C4N atom of the nicotinamide ring, which holds the coenzyme in the active “hydride transfer” conformation in the structure of the enzyme with NADP+ (PDB entry 2O2Q). This bond was shown to be an indicator of the oxidation state of the coenzyme [10, 23]. The same adduct was predicted by quantum chemical calculations for ALDH2 [24]. Meanwhile, a similar adduct of PaBADH with the C2N atom of the nicotinamide ring was observed in complex with NADPH and is, in the authors' opinion, responsible for the reversible inhibition of the enzyme by NADPH [21, 22]. In the other structures of AlDHs with the coenzyme in the “hydride transfer” conformation, the sulfur-carbon covalent bond was not observed.

The mechanism of action of AlDHs has been studied only for the enzymes from mesophilic organisms. In the present work, we report the structural and functional characterization of the first thermostable archaeal dehydrogenase (AlDHPyr1147). The gene encoding AlDHPyr1147 was found in the genome of the archaeon* Pyrobaculum sp.1860*. The hyperthermophilic crenarchaeon* Pyrobaculum sp.1860* is a member of the ancient order Thermoproteales, and it grows optimally at 84°С [25]. We succeeded in structure determination of the apo form, the binary complex with the disordered coenzyme (Holo-1 and Holo-2), the complex with the coenzyme in the ordered “hydride transfer” conformation (Holo-3), and the ternary complex with the coenzyme and the isobutyraldehyde substrate. An analysis of these structures suggests the mechanism of activation of the thiol group, which does not involve the general base catalysis by the catalytic Glu. In addition, we found the blockage of two potential proton relay systems described earlier for AlDHs.

## 2. Materials and Methods

### 2.1. Cloning and Expression of AlDHPyr1147 in* E. coli*

The P186_1147 gene encoding aldehyde dehydrogenase was synthesized by the ATG Service-gene (Russia) and ligated into the pET-21d plasmid (Novagen, Germany).* E. coli* BL21-CodonPlus (DE3)-RIPL cells (Stratagene, USA) transformed with the construct were grown in the LB medium, containing 100 mkg/mL ampicillin, at 37°C until the OD600 value of 0.8, and the expression was induced by 1 mM IPTG. After incubation for 18 h at 25°C, the cells were harvested by centrifugation.

### 2.2. Solubilization, Refolding, and Purification of Recombinant Form of AlDHPyr1147

The* E. coli* cell pellets were suspended in 50 mM Tris-HCl buffer, pH 8.8, containing 1 mM EDTA and 5 mM beta-mercaptoethanol (*β*-ME) (buffer A). The cells were disrupted by double passage through a French pressure cell at 1.3 × 10^8^ Pa. The insoluble fraction containing inclusion bodies was separated by centrifugation at 40,000 ×g for 40 min at 10°C. The insoluble fraction was washed first in buffer A containing 0.5 M NaCl and 0.1% (v/v) Triton X-100, then twice in buffer A containing 0.5 M NaCl, and finally in buffer A containing 0.5 M NaCl and 0.5 M urea. The inclusion bodies were dissolved in 8 M urea prepared in buffer A. Solubilization was carried out at 25°C for 1 h. The insoluble cell fragments were removed at 40,000 ×g for 30 min at 10°C. The supernatant was diluted in 8 M urea in buffer A to the final protein concentration of about 2 mg/mL. The refolding was performed by slow dilution of the protein solution with buffer A to the final urea concentration of 2 M. The resulting protein solution was centrifuged at 40,000 ×g for 30 min at 10°C and then filtered through a 0.45 *μ*m pore size filter (Millipore). The protein solution in 2 M urea was loaded on a Source 30 Q column (GE Healthcare) that has been equilibrated with buffer A. After loading, the column was washed with ten column volumes of buffer A. The fractions were eluted with a linear NaCl gradient from 0 to 500 mM. The homogeneity and purity of the fractions were determined by 12% (w/v) SDS-PAGE with Coomassie Brilliant Blue staining. Protein concentrations were determined by the Bradford method. Refolded AlDHPyr1147 was concentrated and further purified using a Superdex200 10/300 GL column (GE Healthcare) preequilibrated with 50 mM HEPES, pH 7.5, containing 50 mM NaCl and 5 mM *β*-ME (buffer B). The purified enzyme was concentrated and stored at −70°C.

The apo form was prepared by heating at 85°C for 10–15 min at a protein concentration of not more than 0.5 mg/mL in buffer B followed by concentration, centrifugation, and gel filtration at room temperature.

Analytical size-exclusion chromatography was carried out on a 10/300 GL column (GE Healthcare) preequilibrated with 50 mM sodium pyrophosphate buffer, pH 8.8, containing 100 mM NaCl (buffer C) at 22°С.

### 2.3. Enzyme Assay

The AlDHPyr1147 activity was assayed spectrophotometrically at 60°C, unless stated otherwise. The NADP+ reduction was followed by absorbance changes at 340 nm on an Evolution 300 spectrophotometer (Thermo Scientific) using a thermostated cuvette holder equipped with a Peltier element. The reaction mixture (1 mL) contained buffer C, 0.3 mM NADP+, 9 mM isobutyraldehyde, and 10–15 *μ*g protein. The AlDHPyr1147 activity unit was defined as the reduction of 1 *μ*mol NADP+ per minute based on the absorption coefficient for NADPH (6.22 mM^−1^ cm^−1^). The correction for the temperature-dependent spontaneous nonenzymatic reaction with aldehyde substrates was applied. The reported enzyme activities were always linearly proportional to the amount of the enzyme added. The enzyme concentration was determined spectrophotometrically [26].

The kinetic parameters *K*_*m*_ and *V*_max_ of AlDHPyr1147 were calculated from duplicate or triplicate measurements by the Michaelis–Menten equation using the OriginPro 8 program. The turnover number (*k*_cat_, s^−1^) was calculated using the subunit molecular weight of 54.39 kDa.

### 2.4. Temperature and pH Dependence and NAD+ and NADPH Effects

The temperature dependence of the AlDHPyr1147 activity was measured between 45 and 85°C in buffer C containing 0.30 mM NADP+, 9 mM isobutyraldehyde, and 10–15 *μ*g protein per mL. The pH dependence of the enzyme activity was measured between pH 8.0 and pH 10.5 at 60°C with either sodium pyrophosphate or Gly-NaOH buffers (each at 50 mM) containing 100 mM NaCl, 0.3 mM NADP+, 9 mM isobutyraldehyde, and 10–15 *μ*g protein per mL. The effects of NAD+ and NADPH on the AlDHPyr1147 activity were tested by standard assay.

### 2.5. Crystallization, Data Collection, and Processing

Crystals of AlDHPyr1147 as isolated (Holo-1) were grown by the modified capillary counterdiffusion technique [27, 28] in microgravity within six weeks. Crystals of the apo form of AlDHPyr1147 (apo), the complex with NADP+ (Holo-2), and the ternary complex with NADP+ and the substrate were obtained by the hanging-drop vapor-diffusion method in Linbro plates (Hampton Research) at 20°C. Drops consisted of 1 *µ*L of a protein solution and 1 *µ*L of a reservoir solution of different compositions for different models (Table 1). The crystals of Holo-2 and the ternary complex were obtained by cocrystallization. Crystals suitable for X-ray diffraction were grown in ten to fourteen days. Holo-3 was obtained by soaking crystals of the apo form in excess of NADP+ and isobutyraldehyde. Unfortunately, our attempts to obtain crystals of the binary complex with NADPH suitable for X-ray diffraction by either cocrystallization or soaking failed.

The X-ray diffraction data from a crystal of the apo form were collected at the Kurchatov SNC beamline using a MAR CCD 130 mm detector. The data were processed and reduced with XDS [29] and scaled with XSCALE [29]. The X-ray diffraction data from crystals of Holo-1 and Holo-3 were collected at the Spring-8 beamline BL41XU using a MARMOSAIC 225 mm CCD detector. The data were reduced and scaled with the HKL-2000 software package [30]. The X-ray diffraction data from crystals of Holo-2 and the ternary complex were collected at the ESRF beamline ID23 using a DECTRIS PILATUS 6M-F detector. The data were processed, reduced, and scaled with XDS [29]. All crystals were briefly soaked in mother liquor containing 20% glycerol and subsequently flash-cooled in a cold gas stream of nitrogen at 100 K. The experimental details and the data collection statistics are given in Table 2.

### 2.6. Structure Solution and Refinement

The structure of AlDHPyr1147 was solved using the program Molrep [31]. The solution was obtained using as the starting model the 2.4 Å resolution structure of AlDHPyr1147 solved earlier (PDB entry 4H73). The water molecules were removed from the initial model. The further refinement of the model was done using the program Phenix.refine [32]. In all the structures determined in the present study, there are eight identical subunits of the enzyme per asymmetric unit. The coordinates and the individual* B*-factor were refined for each atom. For residues with multiple conformations, the occupancies were refined as well. The visual inspection of electron density maps and manual corrections of the model were performed using Coot [33]. The geometry of the model was analyzed with the Molprobity server (http://molprobity.biochem.duke.edu/). The summary of refinement statistics and the model quality indicators are given in Table 2. The PDB entries of the refined structures are presented in Table 2. The figures were prepared using the Pymol program (http://www.pymol.org/).

## 3. Results and Discussion

### 3.1. Expression, Purification, and Enzymatic Characterization of AlDHPyr1147

Recombinant aldehyde dehydrogenase AlDHPyr1147 was obtained by heterological expression of the synthetic gene P186_1147 in* E. coli*. The gene was synthesized without codon modifications. The tag-free form of AlDHPyr1147 was produced in* E. сoli* in an insoluble form and was sequentially washed, solubilized, and purified to homogeneity. According to gel filtration, AlDHPyr1147 is a tetramer. The kinetic data showed that AlDHPyr1147 is strictly specific for NADP+ and is active with aliphatic aldehydes (Table 3). It was not possible to determine whether AlDHPyr1147 has an activity with phenylacetaldehyde and chlorobenzaldehyde due to extremely high level of spontaneous nonenzymatic reaction. No activity was also detected with betaine aldehyde and succinic semialdehyde.

**Figure 1:**
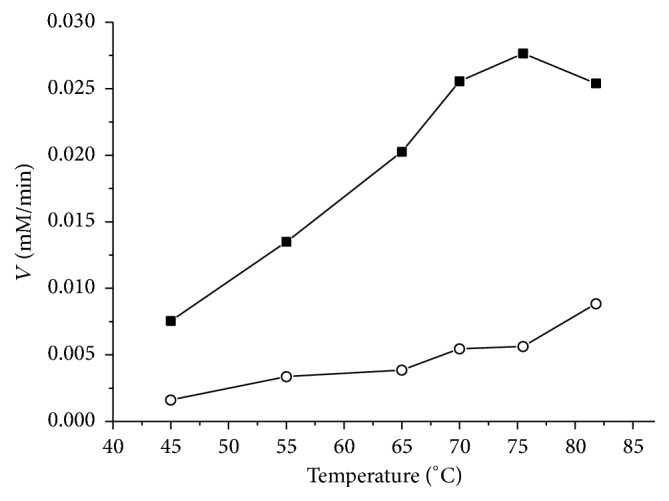
Temperature dependence of the initial rate of the oxidation of isobutyraldehyde with AlDHPyr1147 corrected for the spontaneous nonenzymatic reaction (■). The reaction was initiated by the addition of 10 *μ*g of the enzyme to the reaction mixture in standard assay. (*ο*) The initial rate of the spontaneous nonenzymatic reaction.

The optimum pH range for the oxidation of isobutyraldehyde is 8.5–9.5. The highest rate of isobutyraldehyde oxidation was observed at 75–80°С (Figure 1). However, since the nonenzymatic reaction involving NADP+ and aldehydes becomes significant at these temperatures, the standard assay was developed at 60°С. The Michaelis constant for NADP+ is 20.9 ± 0.1 *μ*M. For comparison, *K*_*m*_ for C_t_-FDH and of homologous dehydrogenase from* S. mutans* (SmAlDH) are 2.0 and 24.5 *μ*M, respectively, at 20°С [34, 35]. The value of *K*_*m*_ of AlDHPyr1147 for NADP+ falls in the *K*_*m*_ range from 1.4 to 210 *μ*М for NADP-dependent AlDHs (EC 1.2.1.4). Neither NADPH nor NAD+ is an efficient competitive inhibitor. Thus, the addition of 0.08 mМ NADPH to a standard reaction mixture (0.084 *μ*М enzyme and 0.3 mM NADP+) leads only to a 20% decrease in activity. The addition of 0.3 mM NAD+ under the same conditions does not change the rate of the standard reaction.

### 3.2. NADP+ and NADPH Binding

**Figure 2:**
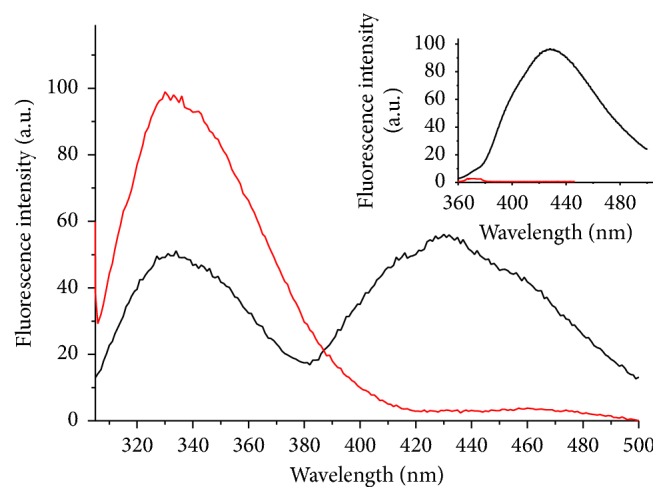
Fluorescence spectra of 0.10 mg/mL AlDHPyr1147 at room temperature: AlDHPyr1147 as isolated (black line), the same enzyme after heating up to 85°C and subsequent cooling to room temperature (red line). The excitation is at 297 and 330 nm (in the inset).

Figure 2 presents the tryptophan fluorescence spectrum of AlDHPyr1147 as isolated. At an excitation wavelength of 297 nm, the fluorescence spectrum shows two peaks with maxima at 332 (tryptophan fluorescence) and 430 nm (enzyme-bound NADPH fluorescence). At an excitation wavelength of 330 nm, the spectrum shows one peak with a maximum at 430 nm (the inset) corresponding to the enzyme-bound NADPH fluorescence [36]. The heating of the enzyme to 85°С followed by cooling leads to the disappearance of the peak at 430 nm under excitation both at 297 and at 330 nm; that is, the coenzyme dissociates from the enzyme, resulting in the conversion of AlDHPyr1147 to the apo form. The spectrophotometric analysis of low-molecular-weight components after the separation of the heated protein using Amicon Ultra centrifugal filter devices (Millipore) demonstrated the presence of both NADP+ and NADPH in the filtrate. The concentrations of both forms of the coenzyme were estimated using the extinction coefficients of 1.8 × 10^4^ M^−1^ cm^−1^ (NADP+ at 260 nm), 1.5 × 10^4^ M^−1^ cm^−1^ (NADPH at 260 nm), and 6.22 × 10^3^ M^−1^ cm^−1^ (NADPH at 340 nm) [10]. The percentage of NADPH in the filtrate was 30%. It should be noted that the dialysis against 1 M NaCl for 4 days led only to the partial removal of the coenzyme (based on the fluorescence spectra). Thus, AlDHPyr1147 is isolated in complex with coenzyme.

Therefore, in subsequent experiments, the apo form was prepared by heating an enzyme solution at a concentration not more than 0.5 mg/mL at 85°С for 10–15 min followed by gel filtration (at a higher enzyme concentration, substantial aggregation was observed). According to gel filtration, the apo form of AlDHPyr1147 is also a tetramer. In standard assay, the activity of the apo form of AlDHPyr1147 thus prepared is similar to that of AlDHPyr1147 as isolated.

The dissociation constant of the binary complex measured by spectrofluorimetric titration of the apo form of AlDHPyr1147 with NADP+ (see Figures S1 and S2 in Supplementary Material available online at http://dx.doi.org/10.1155/2016/9127857) is 0.60 ± 0.08 *μ*M at 60°С for a specified number of binding sites in the tetramer equal to four (*n* = 4). The polynomial approximation with unknown number of binding sites in the tetramer gave lower *K*_*d*_ = 0.13 ± 0.03 *μ*M and *n* = 7. For comparison, the dissociation constants of the binary complexes of SmAlDH are 2.3 *μ*M for NADP+ and 0.3 *μ*M for NADPH at 25°С [35]. The dissociation constants of the complexes of both enzymes with NADP+ have similar values; that is, at high temperatures, the complex of thermostable AlDHPyr1147 with NADP+ is as stable as the binary complex of the mesophilic homologue. It should be noted that the titration of the apo form of AlDHPyr1147 with NADP+ did not lead to a shift of the fluorescence maximum of the protein spectrum (see Figure S1); that is, the coenzyme binding did not lead to changes in the structure, at least, in the vicinity of tryptophan residues.

The titration of the apo form of the enzyme with NADPH led to unexpected results. Thus, the final protein fluorescence spectrum under excitation at 297 nm at the maximum NADPH concentration differs from the spectrum of AlDHPyr1147 as isolated (Figure 2): a pronounced maximum at 430 nm is absent, while a weak shoulder appears at 455 nm (see Figures S1 and S3a). The intrinsic fluorescence of NADPH changes insignificantly after the addition to the enzyme solution (see Figures S1 and S3b). It looks like the binding to AlDHPyr1147 does not lead to dequenching of NADPH fluorescence [36, 37]. Apparently, the titration of the apo form with NADPH under experimental conditions either does not give rise to the binary complex or affords a complex with NADPH in a conformation different from that of NADPH in the AlDHPyr1147 as isolated. The experimental results did not allow us to calculate the dissociation constant of the binary complex of AlDHPyr1147 with NADPH.

Therefore, AlDHPyr1147 is active thermostable aldehyde dehydrogenase strictly specific for NADP. The kinetic and thermodynamic characteristics of archaeal AlDHPyr1147 in the survival temperature range of the host organism (60°С and higher) are comparable with those of the homologous enzymes from mesophilic organisms. The recombinant form of the enzyme is copurified with the coenzyme, which can be removed by heating. It is unclear why we failed to observe the formation of the complex with NADPH by means of spectrofluorimetric titration. This may be a result of low affinity for the reduced form of the coenzyme or may be a consequence of the unfavorable experimental conditions used because the titration was performed under the conditions optimum for the binding of the oxidized form of the coenzyme. Apparently, the low affinity accounts also for unsuccessful attempts to obtain crystals of the binary complex of AlDHPyr1147 with NADPH using soaking.

### 3.3. Overall Structure of AlDHPyr1147

**Figure 3:**
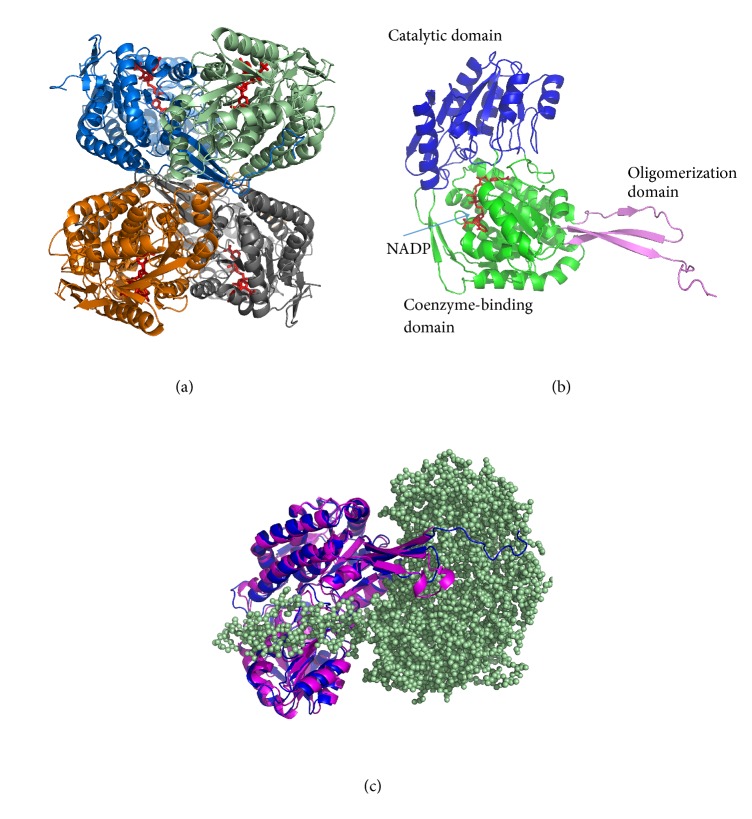
Overall structure of AlDHPyr1147. The coenzyme is in red. (a) The structure of the tetramer. The subunits that form the tetramer are in blue, grey, brown, and pale green. (b) The structure of the subunit. The catalytic, coenzyme-binding, and oligomerization domains are in blue, green, and magenta, respectively. (c) The superposition of the subunit of aldehyde dehydrogenase from* Streptococcus mutants*, PDB entry 2EUH (magenta), and the subunit of AlDHPyr1147 (blue). The second subunit of AlDHPyr1147 that forms a dimer with the first subunit is shown as pale green spheres.

All crystals grown in this study share the same space group P2_1_2_1_2. In all crystal structures of AlDHPyr1147, there are two tetramers per asymmetric unit (Figure 3(a)). Each tetramer is composed of four identical subunits (A, B, C, and D and E, F, G, and H). The structures of the tetramers are very similar in all the crystal structures. For example, the С*α*-atom RMSD between the superimposed tetramers of Holo-1 and Holo-3 is 0.17 Å. The AlDHPyr1147 subunit (Figure 3(b)) adopts the canonical aldehyde dehydrogenase fold and its structure is similar to the structures of the subunits from bacterial AlDHs and human ALDH2 determined earlier. The closest structural analogues of AlDHPyr1147 are benzaldehyde dehydrogenase from* Corynebacterium glutamicum* (PDB entry 3R64, sequence identity 37%, hereinafter BenDH), NADP-dependent dehydrogenase from* S. mutans* (PDB entry 2EUH, sequence identity 35%, hereinafter SmAlDH), and succinic semialdehyde dehydrogenase from* E. coli* (PDB entry 3JZ4, sequence identity 35%, hereinafter SSADH).

The AlDHPyr1147 subunit consists of three domains: the coenzyme-binding domain (residues 1–256, 443–470), the catalytic domain (residues 257–442), and the oligomerization domain (residues 122–144, 470–491). The С-terminus in the oligomerization domain of AlDHPyr1147 is 11 amino acid residues longer compared to the known structures of bacterial AlDHs (Figure 3(c)). These additional C-terminal residues are located close to the surface of the other subunit of the dimer and are involved in hydrogen bonding and hydrophobic interactions between the two subunits, thus strengthening the interaction between them. Arg482 participates in a salt bridge and hydrogen bonds between residues of the two subunits. Phe484, Pro485, Ile486, Pro487, and Leu490 participate in hydrophobic interactions and Ser488 participates in hydrogen bonds between residues of these subunits. The interface surface area between two subunits in the dimer of AlDHPyr1147 calculated with the PISA server (http://www.ebi.ac.uk/pdbe/pisa/) is 3438.6 Å^2^, whereas this parameter value for the closest homologues BenDH, SmAlDH, and SSADH is 1758.7, 2385.7, and 2468.5 Å^2^, correspondingly. The interface surface area calculated for the AlDHPyr1147 model, from which 11 last residues were removed, was 2250.1 Å^2^. The contribution of the last 11 residues to the interface surface area is equal to almost 35%. Thus, the interaction between the subunits in the dimer is significantly strengthened by these additional 11 residues.

An even longer tail at the С-terminus was observed only in human fatty aldehyde dehydrogenase (PDB entry 4QGK, [38]). In the latter structure, the *α*-helix at the C-terminus was shown to cover the substrate channel entrance, acting as a gatekeeper. In the AlDHPyr1147 structure, the tail is shorter and does not contain *α*-helices. Besides, this tail does not close the possible substrate channel and, most likely, only makes it somewhat narrower. Therefore, it cannot be said with certainty whether this elongated tail in the AlDHPyr1147 structure has a functional role apart from the strengthening of the interaction between the subunits in the dimer.

### 3.4. Structures of the Apo Form of AlDHPyr1147 and the Holo-1 Complex of AlDHPyr1147

It is evident from the preliminary study that AlDHPyr1147 as isolated is a complex with the coenzyme. Holo-1 is a model of its crystal structure. The superposition of the subunits of Holo-1 and the apo form of the enzyme shows that the removal of the coenzyme molecule does not cause movements or conformational changes in the secondary structure elements of the coenzyme-binding domain. The С*α*-atom RMSD between the superimposed subunits of Holo-1 and apo is 0.30 Å. A comparison of the apo forms and the complexes with the coenzyme for the AlDH structures shows that, in most cases, the structure of the apo form is very similar to that of the enzyme in complex with the coenzyme. For example, this was shown for betaine aldehyde dehydrogenase [17], mitochondrial aldehyde dehydrogenase [39], and C_t_-FDH [6]. A slight difference in the structures of the apo form and the enzyme in complex with the coenzyme was observed for lactaldehyde dehydrogenase from* E. coli* [19], the differences being found only in one loop of the catalytic domain, whereas the coenzyme-binding domain in the apo form is identical to that in complex with the coenzyme. The SmAlDH structure [7] is the only one, in which the structure of the coenzyme-binding domain changes upon the coenzyme binding (the changes are observed only in one loop). It should also be noted that the *α*-helix involved in the adenine moiety binding was found to be disordered in the apo form of the C_t_-FDH mutant, whereas this helix in the binary complex with the coenzyme is highly ordered [10]. The authors suggested that the *α*-helix is disordered in the native structure as well; however, this hypothesis has not yet been experimentally confirmed.

**Figure 4:**
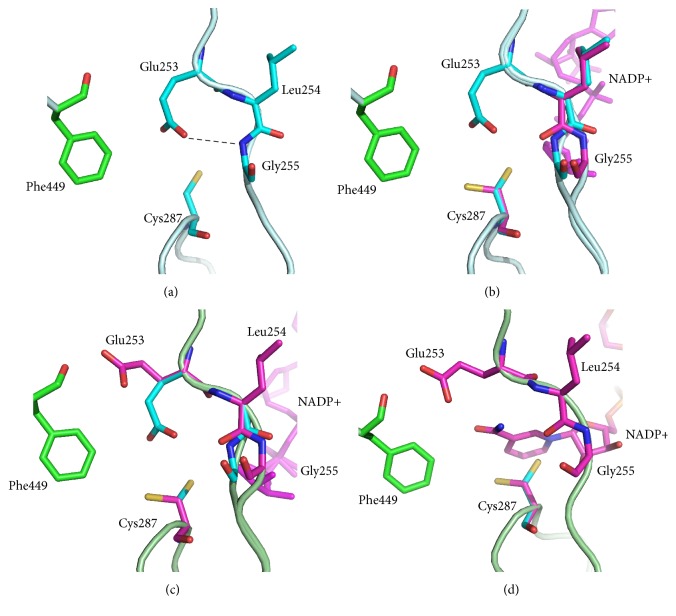
Conformations of the active site residues in (a) the apo form, (b) Holo-1, (c) Holo-2, and (d) Holo-3. The coenzyme is in magenta. When the coenzyme is present in the active site, the Glu253, Cys287, and Gly255 residues are in conformations shown in magenta. When there is no coenzyme in the active site, the Glu253, Cys287, and Gly255 residues are in conformations shown in cyan. Hydrogen bonds are shown by dashed lines.

In the apo structure, conformational changes are observed only for the residues of the active site as compared to the Holo-1 structure. The side chains of the catalytic Cys287 and Glu253 residues in apo are directed inward in the coenzyme-binding pocket (Figure 4(a)). The distance between SG of Cys287 and the nearest atom of the Glu253 residue (OE1 or OE2 in different subunits) varies from 3.12 to 3.32 Ǻ. The OE2 atom of Glu253 forms a hydrogen bond with the nitrogen atom of Gly255.

**Figure 5:**
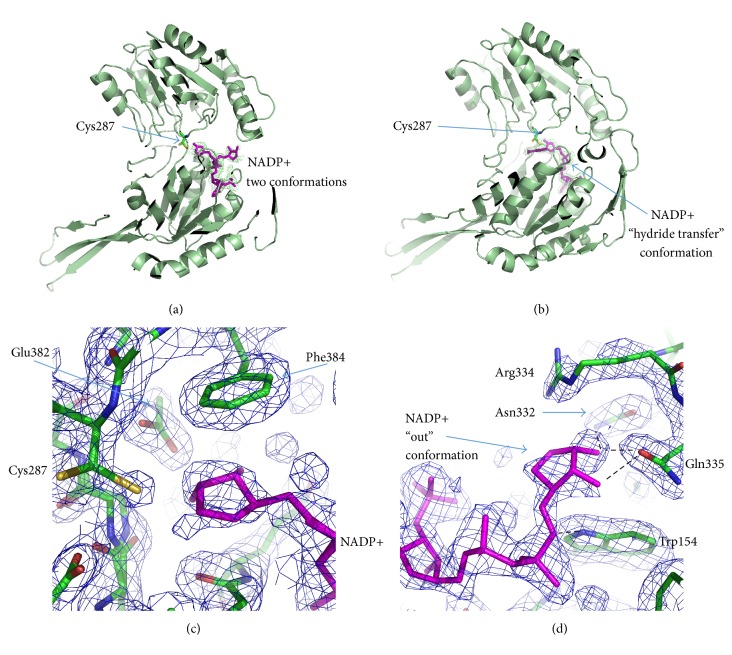
Different conformations of the coenzyme in the complexes with AlDHPyr1147. Coenzyme is in magenta, and AlDHPyr1147 is in green. (a) The coenzyme is in two conformations in the Holo-1 and Holo-2 complexes. The omit *F*_obs_ − *F*_calc_ electron density map for the model Holo-2 around the coenzyme is contoured at 2.7*σ* and colored green. (b) NADP+ is in the “hydride transfer” conformation in the Holo-3 model. The omit *F*_obs_ − *F*_calc_ electron density map for the model Holo-3 around the coenzyme is contoured at 3*σ* and colored green. ((c) and (d)) The 2*F*_obs_ − *F*_calc_ electron density maps for the AlDHPyr1147 model contoured at 1*σ* and colored blue. Hydrogen bonds are shown by dashed lines. (c) An enlarged view of coenzyme in the “hydride transfer” conformation in the active site of the Holo-1 model. (d) An enlarged view of coenzyme in the “out” conformation in the Holo-1 model.

In the Holo-1 structure, the coenzyme adopts the “out” and “hydride transfer” conformations with approximately equal occupancies in all subunits except B (Figure 5(a), Table S1). In subunit B, the coenzyme is only in the “hydride transfer” conformation. In all subunits, the nicotinamide ring of the coenzyme is disordered and the nicotinamide part of the coenzyme is stabilized in the “hydride transfer” conformation by a hydrogen bond between the O2D atom and Glu382 and by a stacking interaction between the nicotinamide ribose and Phe384 (Figure 5(c)). In the “out” conformation, the nicotinamide ribose forms hydrogen bonds with Trp154, Asn332, and Gln335 (Figure 5(d)). In the Holo-1 structure, as opposed to the structure of the apo form, the active site residues have multiple conformations (Figure 4(b)). Cys287 and the main chain for residues 254-255 are in double conformations, while Glu253 has only one conformation with partial occupancy. The electron density corresponding to the second possible conformation of Glu253 was too weak and did not allow building it. When the coenzyme is in the “hydride transfer” conformation, the Glu253 residue is disordered, and the Cys287 side chain is directed away from the coenzyme-binding pocket and forms a hydrogen bond with a water molecule (HOH 3230 in subunit А), which, in turn, forms a hydrogen bond with ND2 of Asn155. When the coenzyme is in the “out” conformation, both catalytic residues are directed towards the center of the coenzyme-binding pocket, like in the apo structure, in which the distances between SG of Cys287 and OE1 of Glu253 vary from 3.12 to 3.82 Å in two different subunits. This attests to the presence of a hydrogen bond between these atoms. Like in the apo form, the Glu253 residue forms a hydrogen bond with the nitrogen atom of Gly255.

In the AlDHPyr1147 structures determined in this study, the adenine moiety of the coenzyme is well ordered, whereas the nicotinamide moiety of the coenzyme is partially disordered. The coenzyme adopts the “hydride transfer” and “out” conformations. The “hydrolysis” conformation of the coenzyme is not observed in the AlDHPyr1147 structures (Table S1).

### 3.5. Structure of the Active Site in the Holo-2 and Holo-3 Models of AlDHPyr1147 in Complex with the NADP+

Crystals of the binary complexes Holo-2 and Holo-3 were obtained under different conditions (Table 1). The crystal of Holo-2 was grown by cocrystallization of the apo form with NADP+, whereas the crystal of Holo-3 was prepared by soaking crystals of the apo form in a solution of NADP+ and isobutyraldehyde. In Holo-2, the conformations of the coenzyme and the active site residues are very similar to those in the Holo-1 structure (Table S1, Figure 5(a)). The main difference is in the conformation of Glu253. In Holo-2, Glu253 has two conformations, the “inside” and “intermediate” conformations (Figure 4(c)). In the latter conformation, the *γ*-carboxyl group of Glu253 is directed away from the active site and is at a distance of 2.7 Ǻ from O_Phe449. In this conformation, Glu253 is not involved in a hydrogen bond with nitrogen of Gly255, and the carbonyl oxygen of Leu254 is rotated by 180°.

In the Holo-3 model, the nicotinamide ring is clearly seen in electron density maps (Figure 5(b)). The substrate is not present in the active site, although its addition via soaking seems to order the nicotinamide ring. The ordered nicotinamide part of NADP+ in the active site corresponds to such conformations of Cys287 and Glu253 in which the side chains are directed away from the nicotinamide moiety and do not form hydrogen bonds with each other. Glu253 is observed only in the “intermediate” conformation; the peptide bond between Leu254 and Gly255 is oriented in such a way as to form the hydrogen bond between O_Leu254 and N7N of NADP+ (Figure 4(d)); N7N is also within 3.4 Ǻ of the carbonyl oxygen of Leu254. Therefore, the ordered nicotinamide ring in the active site is held in place by one hydrogen bond and, apparently, by an electrostatic interaction with the thiolate anion of Cys287. In the structures of ALDH2, PaBADH, and C_t_-FDH, the nicotinamide ring is also held in place by a single hydrogen bond [5, 6, 16, 22]. The authors suggest that van der Waals interactions make the major contribution to stabilization of the nicotinamide part. When the nicotinamide ring of the coenzyme is disordered, the thiol group of Cys287 is directed inward in the active site.

It should be noted that, as follows from the crystallization conditions for the Holo-2 structure, the coenzyme is in the oxidized form. Earlier, it has been discussed [19] that the “out” conformation may correspond both to the NADP+ binding to AlDH and to the release of NADPH.

While the nicotinamide part of the coenzyme is flexible and adopts different conformations, the adenine part of the coenzyme is well ordered in all complexes of AlDHPyr1147. The adenine part of the coenzyme is fixed in a narrow cleft between helix 211–230 and helix 230–244 (Supplementary Figure S4); the 5′-phosphate of the adenosine ribose forms hydrogen bonds with Lys178, Ser181, and Gly211. Superposition of the Holo-2 and the complexes of SmAIDH and SSADH with NADP+ reveals the similarity of the position of adenine part. Despite the fact that many residues in the nucleotide binding cleft are not conserved, the number of hydrogen bonds between adenine part of the coenzyme and the protein is almost the same in these structures. For example, Ser231 in SmAIDH and Ser233 in SSADH form a hydrogen bond with O2A atom of the coenzyme. In AlDHPyr1147, Ser is changed for Glu232 and this hydrogen bond does not exist; however, there is a new hydrogen bond between Thr235 and O2A atom, which is absent in the homologous structures. In AlDHPyr1147, the cleft is strengthened by hydrogen bonds between Glu216, water molecules, and Glu238. Moreover, Glu238 and Glu216 form an additional stabilizing water-mediated hydrogen bond with N6A. And finally in AlDHPyr1147 the very rigid fragment Pro210-Gly211–Pro212 seems to strengthen the entrance to the cleft and fixes the position of conserved Gly211. We suggest that the strengthening of the AlDHPyr1147 overall structure and its coenzyme-binding cleft ensures the thermal resistance of the binary complexes.

### 3.6. Structure of the Active Site in AlDHPyr1147 in Complex with the NADP+ and the Substrate: The Substrate Channel Structure

**Figure 6:**
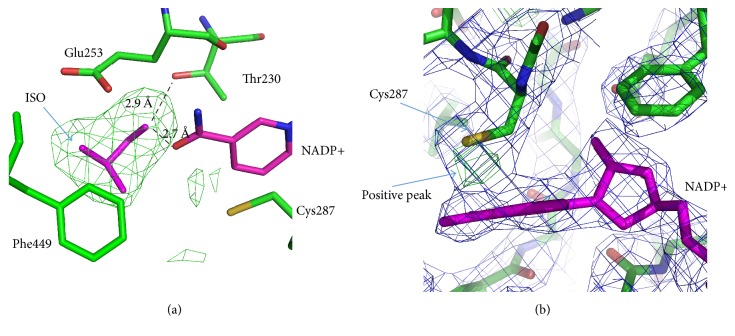
Active site in the complex of AlDHPyr1147 with isobutyraldehyde (ISO) and NADP+. The active site residues are in green. NADP+ and isobutyraldehyde are in magenta. The 2*F*_obs_ − *F*_calc_ electron density map for the AlDHPyr1147 model is contoured at 1*σ* and colored blue. (a) The omit *F*_obs_ − *F*_calc_ electron density map for the model of the tertiary complex around the region of the substrate. The map is colored green and contoured at 3*σ*. (b) Positive electron density in the *F*_obs_ − *F*_calc_ electron density map for AlDHPyr1147, which is indicative of the possible second conformation of Cys287. The *F*_obs_ − *F*_calc_ electron density map for the AlDHPyr1147 model is contoured at +3.2*σ* and colored green. Hydrogen bonds are shown by dashed lines.

On the omit-map calculated for the model of the ternary complex without substrate, there were strong positive densities at the same place in the active sites of all six monomers (A, B, C, D, G, and H). These densities were identified at the places where normally water molecules were observed in the complexes of AIDHPyr1147 with the coenzyme. The densities had similar shapes in different monomers, and the isobutyraldehyde substrate fitted its well. Therefore, we interpreted this density as the substrate isobutyraldehyde (Figure 6(a)). The oxygen atom of the substrate forms hydrogen bonds with the O7N atom of the coenzyme and the OG1 atom of Thr230. The substrate molecule is surrounded by the Ile160, Lys163, Lys164, Phe449, Glu443, and Glu460 residues, the hydrophobic parts of the Lys and Glu side chains turning to the substrate.

The superposition of the ternary complex of AlDHPyr1147 with the other known complexes of AlDHs with the coenzyme and the substrate shows that the isobutyraldehyde molecule is displaced by approximately 3 Å from the substrate position in the complexes of other AlDHs. The isobutyraldehyde molecule is located at the bottom of the substrate channel at a hydrogen-bond distance from the coenzyme. However, it would not be appropriate to consider this structure as a productive enzyme-substrate complex or an intermediate compound since the substrate molecule is located at a too large distance from the catalytic Cys287 (the distance between the substrate and SG of Cys287 is 5.74 Å). A similar situation was observed, for example, for 6-semialdehyde dehydrogenase from* Pseudomonas fluorescens* in complexes with the coenzyme and the substrate (PDB entries 4I2R and 4I25, [40]).

The structure of the active site in the ternary complex is similar to that in the Holo-3 model (Figure 4(d)), and NADP+ is fully ordered in the “hydride transfer” conformation. This is the only crystal structure of AlDHPyr1147 in which the occupancy for the nicotinamide ring is 1.0. The Cys287 residue has only one (rather than two) conformation, in which the side chain is directed away from the coenzyme. The other active site residues have the same conformations as in the Holo-3 structure. It can be concluded that substrate binding leads to ordering of the nicotinamide ring and the active site in general.

The observed high positive density in *F*_obs_ − *F*_calc_ electron density maps between the nicotinamide ring and the SG atom (Figure 6(b)) attests to the probable second conformation of Cys287, the position of which is similar to the major conformation. In the second conformation, Cys287 can be involved in the formation of a covalent bond with the С4N atom of the coenzyme (like in the C_t_-FDH model [6]). The distance between the positive electron density peak in the *F*_obs_ − *F*_calc_ map and the С4N atom of the coenzyme is about 1.5 Å. In C_t_-FDH, the same distance in the cysteine-nicotinamide adduct for the cysteine in a conformation different from those directed towards and away from the coenzyme-binding pocket is about 1.6 Å. However, the resolution for the structure is not sufficiently high to reliably determine whether the cysteine-nicotinamide adduct is present in the AlDHPyr1147 structure.

**Figure 7:**
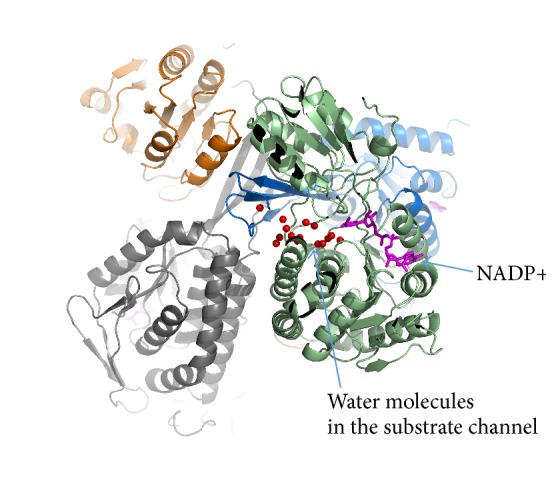
A putative substrate channel. The subunits that form the tetramer are shown in blue, grey, brown, and light green. The coenzyme is in magenta. The water molecules in the substrate channel are shown as red spheres.

Based on the structural analysis, several research groups hypothesized [5, 6, 18] that, in the AlDH structures, the substrate reaches the active site through a separate substrate channel, which comes to the surface of the enzyme opposite to the coenzyme entrance. The AlDHPyr1147 structure also contains a channel, via which the substrate can access the active site. The substrate channel in AlDHPyr1147 is composed mostly of residues of one subunit and, at the exit from the channel, of a few residues of another subunit that forms a dimer with the first subunit. This channel is funnel-shaped and it becomes narrower at the proximity of the active site. The substrate channel is lined by both polar and hydrophobic residues (Arg101, Lys102, Trp104, Gly105, Val108, Phe109, Arg112, Tyr156, Ser159, Ile160, Lys163, Phe277, Phe281, Ile286, Phe441, Leu442, and Glu443) and by the residues of another subunit (Asp132, Ser133, Glu134, Ser135, Thr481, Arg482, Arg483, Phe484, and Pro485) at the exit. In all the models of the binary complex, the substrate channel is occupied by water molecules (Figure 7).

### 3.7. Analysis of Possible Proton Release Pathways

The accepted mechanism of aldehyde oxidation with AlDHs involves the proton relay from the catalytic Glu, which accepts the proton in the first acylation step from the catalytic Cys. Two potential proton relay systems in the AlDH structures have been proposed [5, 6, 8]. Thus, the catalytic Glu in the C_t_-FDH structure (PDB entry 2O2R) directly contacts a continuous chain of water molecules, which exits into a narrow channel between two subunits [6]. However, other structures, for example, PaBADH (PDB entry 2WME), do not contain a continuous water chain from the catalytic Glu. González-Segura and coworkers [8] suggested that the proton transfer in the PaBADH structure occurs from the catalytic Glu to Glu464 (the residue numbering used in 2WME; in the AlDHPyr1147 structure, Glu460) and then via a continuous water chain from Glu464 into a narrow channel between two subunits.

A comparison of the AlDHPyr1147, C_t_-FDH, and PaBADH structures shows that the crystal structure of AlDHPyr1147 does not contain a continuous water chain (except water in the substrate channel) that could link either Glu253 or Glu460 to the channel between the subunits. The water chain directly from Glu253 is interrupted by the Arg459 residue, which is tightly held in place by three hydrogen bonds with adjacent residues (in PaBADH, this channel is blocked in a similar fashion). The water chain from the residue Glu460 towards the channel between the subunits in AlDHPyr1147 is interrupted by Glu468. Unlike Arg459, Glu468 can be potentially involved in the proton transfer via a hydrogen bond with Thr251. However, this chain cannot exist because Glu253 and water molecules in this system form hydrogen bonds with different oxygen atoms of the *γ*-carboxyl group of Glu460; that is, the proton is accepted by one oxygen atom but can be abstracted only from another one (the same situation is observed in the structure of dehydrogenase from* B. xenovorans*, PDB entry 2VRO). As opposed to AlDHPyr1147, the same oxygen atom of Glu464 is present in the channel at hydrogen-bond distances from both catalytic Glu and a water molecule in the PaBADH structure. Therefore, the proton relay in AlDHPyr1147 through the Glu460 residue is impossible. In our opinion, the proton relay from Glu253 in AlDHPyr1147 can occur through the substrate channel, which forms contacts with both Glu253 and Glu460.

### 3.8. Communications between Mobile Elements in the Active Site of AlDHPyr1147

In the AlDHPyr1147 structures, the catalytic Cys is found in two stable conformations, in which the side chain is directed towards or away from the nicotinamide moiety of the bound coenzyme. Only in the latter conformation is the thiol group directed towards the substrate channel; and only in the former inactive conformation is the thiol group of Cys involved in hydrogen bonds with the catalytic Glu. The formation of such hydrogen bonds is indicative of similar values of pKa for the side chains of the residues and sometimes is interpreted in terms of general base catalysis of the activation of the catalytic Cys from the catalytic Glu [5, 22]. Another way of activation of the catalytic Cys related to the local structural rearrangement upon the coenzyme binding has been discussed in [7]. The authors suggested that the coenzyme binding leads to rotation of Cys and its activation, a decrease in pKa followed by the formation of a thiolate ion, as a result of the interaction with the peptide nitrogen atom of Сys and adjacent Thr. In the ternary complex of AlDHPyr1147, the SG atom of Cys287 in the active conformation is at a hydrogen-bond distance from the peptide nitrogen atom of Thr288, and apparently this hydrogen bond can lead to a decrease in pKa of Cys. Our data do not contradict the decrease in pKa of the thiol group of Cys due to the interaction with the nicotinamide ring of NADP+.

In the “inside” conformation, Glu253 forms, apart from a weak hydrogen bond with Cys287, a strong hydrogen bond with HN_Gly255, which is indicative of the deprotonated state of its carboxyl group. In the “intermediate” conformation, Glu253 forms a hydrogen bond with the carbonyl oxygen atom of Phe449. This attests to the protonated state of its side chain and increased pKa. Based on the analysis of the models determined in the present study, it can be hypothesized that Glu253, when it moves from the “inside” to “intermediate” conformation, can accept a proton that is released upon deprotonation of S-H bond of Cys. The factors responsible for an increase in pKa of Glu in the “intermediate” conformation of AlDHPyr1147 are unclear. This fact might be attributed to the environment of this residue because it is different in the “inside” and “intermediate” conformations of Glu253.

Based on the structural data, some conclusions can be drawn concerning the acylation step of aldehyde oxidation by AlDHPyr1147. The substrate and the coenzyme enter into the active site from the opposite sides. The substrate moves through a special channel, which is open regardless of the presence of the coenzyme. In the beginning of the reaction, NADP+ is in the “hydride transfer” or “out” conformation with the disordered nicotinamide ring, and the side chains of the catalytic Cys287 and Glu253 residues are directed inward in the coenzyme-binding pocket and are hydrogen-bonded. The transition to the active conformation of Сys287 (rotation of its thiol group) is accompanied by the ordering of the nicotinamide ring of the coenzyme in the active site and rotation of the Glu253 side chain. In the active conformation, the thiol group of Cys287 is oriented into the substrate channel. The proton that is released can be accepted by the side chain of Glu253, resulting in the transition of the latter to the “intermediate” conformation stabilized by a hydrogen bond with the O_Phe449 atom. This makes it possible to remove the proton from the active site. We suggest that the proton relay from Glu253 occurs via the substrate channel. The proton relay from Cys287 to the substrate channel, which is not mediated by Glu253, cannot be ruled out as well since the geometry of the active site allows for this process to occur.

## 4. Concluding Remarks

The following conclusions can be drawn: (1) AlDHPyr1147 is thermostable aldehyde dehydrogenase specific for aliphatic aldehydes having the pH optimum in the alkaline region; the affinity of AlDHPyr1147 for the NADP+ is similar to that in mesophilic homologues; (2) the active stable form of AlDHPyr1147 is a tetramer; the three-dimensional structure of each subunit is similar to that of the subunits of AlDHs characterized earlier; (3) the main difference between the overall structure of thermostable ALDHPyr1147 and the structures of homologous mesophilic AlDHs is that it has the longer oligomerization domain in the subunit and a larger surface area of the dimer contact in the tetramer; (4) the removal of the coenzyme from AlDHPyr1147 causes no changes in the polypeptide chain fold and no disorder of secondary structure elements; (5) the ordered nicotinamide part in the complex is stabilized by one hydrogen bond with O atom of Leu254 and, probably, by an electrostatic interaction with the thiolate anion of Cys287; (6) the proton release channels proposed earlier for AlDHs are blocked in the AlDHPyr1147 structure; (7) the presence of the substrate in the crystallization solution leads to ordering of the coenzyme in the binary complex.

## Supplementary Material


Supplementary material contains (1) description of the determination of *Kd* values using Scatchard analysis; (2) Figures S1, S2 and S3(a,b) illustrating changes in the emission spectra of apo form of AlDHPyr1147 upon titration with coenzyme at 60°C; (3) Table S1 that lists conformations of the catalytic residues and the coenzyme in the models of AlDHPyr1147; (4) Figure 4S illustrating Nucleotide binding site structure of the binary AlDHPyr1147-NADP+ complex.

## Figures and Tables

**Table 1:** Crystallization conditions for different models of AlDHPyr1147.

Crystals	Composition of protein solution	Composition of reservoir solution
Apo form (*Apo*)	17.7 mg/mL apo form (0.32 mM of monomer) in 50 mM HEPES, pH 7.5, containing 50 mM NaCl and 5 mM *β*-ME	0.1 М imidazole, pH 6.5, and 1.0 M sodium acetate trihydrate

AlDHPyr1147 as isolated (*Holo-1*)	17 mg/mL AlDHPyr1147 *as isolated* in 50 mM HEPES, pH 7.5, containing 50 mM NaCl and 5 mM *β*-ME	0.01 M nickel(II) chloride, 0.1 M Tris, pH 8.5, 1.4 M lithium sulfate, and 50 mM NaCl

Complex with NADP+ (*Holo-2*)	Cocrystallization of 17.7 mg/mL apo form with 27 mM NADP+ in 50 mM HEPES, pH 7.5, containing 50 mM NaCl and 5 mM *β*-ME	0.1 М imidazole, pH 6.5, and 1.0 M sodium acetate trihydrate

Complex with NADP+ (*Holo-3*)	Soaking crystals of the apo form in excess of NADP+ and isobutyraldehyde for 40 min at 25°C	

Ternary complex with NADP+ and substrate (*ternary complex*)	Cocrystallization of 17.7 mg/mL apo form with 27 mM NADP+ and 400 mM isobutyraldehyde in 50 mM HEPES, pH 7.5, containing 50 mM NaCl and 5 mM *β*-ME	0.1 М imidazole, pH 6.5, and 1.0 M sodium acetate trihydrate

**Table 2:** Data collection and refinement statistics. Values in parentheses are for the highest resolution shell.

	Apo	Holo-1	Holo-2	Holo-3	Ternary complex
*PDB entry*	*5EEB*	*5F2C*	*5EUY*	*5EXF*	*5EK6*
*Data collection*					
Space group	P 2_1_ 2_1_ 2	P 2_1_ 2_1_ 2	P 2_1_ 2_1_ 2	P 2_1_ 2_1_ 2	P 2_1_ 2_1_ 2
Cell dimensions					
*a* (Å)	184.24	185.36	184.77	185.59	183.80
*b* (Å)	207.23	208.30	208.05	208.72	207.85
*c* (Å)	164.96	163.81	165.40	164.75	166.44
Temperature (K)	100	100	100	100	100
Wavelength (Å)	0.984	0.800	0.970	1.00	0.972
Crystal-to-detector distance (mm)	168.0	300.0	363.1	300.0	386.9
Oscillation range (°)	1.0	0.5	0.1	0.5	0.1
Number of frames	197	720	1120	720	1450
Resolution (°)	29.77–3.04	29.15–1.90	59.06–2.06	90.97–2.19	59.14–2.66
Completeness (%)	98.9 (85.0)	99.8 (99.0)	99.1 (99.0)	98.2 (97.9)	99.34 (100)
*R*-merge	0.27 (0.76)	0.07 (0.33)	0.10 (0.77)	0.19 (0.73)	0.17 (0.77)
*I*/*σ*	7.7 (2.3)	13.4 (1.6)	8.3 (1.6)	5.3 (1.7)	7.3 (1.8)
Redundancy	7.4	4.0	4.1	4.9	5.4
*Refinement*					
*R*_work_/*R*_free_	0.202/0.256	0.184/0.218	0.196/0.233	0.206/0.245	0.189/0.240
Wilson *B*-factor (Å^2^)	43.5	25.0	35.3	22.0	42.0
Number of atoms					
All	30043	34276	33663	33881	30912
(i) Protein	30043	30690	30355	30382	30054
(ii) NADP+	—	396	408	384	384
(iii) Isobut.	—	—	—	—	30
(iv) Glycerol	—	6	—	6	—
(v) Water molecules	—	3184	2900	3109	444
Average *B*, all atoms (Å^2^)	35.0	28.0	37.0	27.0	40.0
RMS deviations					
(i) Bond lengths	0.010	0.008	0.008	0.008	0.011
(ii) Bond angles	1.274	1.021	1.058	1.071	1.197
Ramachandran plot					
Most favorable (%)	97.43	97.34	96.80	96.24	95.41
Allowed (%)	2.47	2.45	2.86	3.40	3.94
Outliers (%)	0.1	0.21	0.34	0.37	0.65

**Table 3:** Kinetic parameters of AlDHPyr1147 in reactions with different aldehydes. Conditions: 50 mM sodium pyrophosphate buffer, pH 8.8, 100 mM NaCl, and 0.3 mM NADP+, 60°C.

Substrate	*k*_cat_, s^−1^	*K*_*m*_, M	*k*_cat_/*K*_*m*_, s^−1^ M^−1^
Isobutyraldehyde	4.1 ± 0.4	0.020 ± 0.003	200 ± 50
Propionaldehyde	2.9 ± 0.16	0.021 ± 0.0025	140 ± 30
Glyceraldehyde	1.2 ± 0.2	0.015 ± 0.006	80 ± 40

## Abbreviation

AlDH: Nonphosphorylating hydrolytic aldehyde dehydrogenase
AlDHPyr1147: Aldehyde dehydrogenase from archaeon* Pyrobaculum sp.1860*
ALDH2: Human aldehyde dehydrogenase
BenDH: Benzaldehyde dehydrogenase from* Corynebacterium glutamicum*
C_t_-FDH: 10-Formyltetrahydrofolate dehydrogenase from* Rattus norvegicus*
PaBADH: Betaine aldehyde dehydrogenase from* Pseudomonas aeruginosa*
SSADH: Succinic semialdehyde dehydrogenase from* Escherichia coli*
SmAlDH: NADP-dependent dehydrogenase from* Streptococcus mutans*.

## References

[1] Valenzuela-Soto E. M., Munoz-Clares R. A. (1993). Betaine-aldehyde dehydrogenase from leaves of *Amaranthus hypochondriacus* L. exhibits an Iso Ordered Bi Bi steady state mechanism. *Journal of Biological Chemistry*.

[2] Sidhu R. S., Blair A. H. (1975). Human liver aldehyde dehydrogenase. Kinetics of aldehyde oxidation. *Journal of Biological Chemistry*.

[3] Feldman R. I., Weiner H. (1972). Horse liver aldehyde dehydrogenase. II. Kinetics and mechanistic implications of the dehydrogenase and esterase activity. *The Journal of Biological Chemistry*.

[4] Marchal S., Cobessi D., Rahuel-Clermont S., Tête-Favier F., Aubry A., Branlant G. (2001). Chemical mechanism and substrate binding sites of NADP-dependent aldehyde dehydrogenase from *Streptococcus mutans*. *Chemico-Biological Interactions*.

[5] Muñoz-Clares R. A., González-Segura L., Díaz-Sánchez Á. G. (2011). Crystallographic evidence for active-site dynamics in the hydrolytic aldehyde dehydrogenases. Implications for the deacylation step of the catalyzed reaction. *Chemico-Biological Interactions*.

[6] Tsybovsky Y., Donato H., Krupenko N. I., Davies C., Krupenko S. A. (2007). Crystal structures of the carboxyl terminal domain of rat 10-formyltetrahydrofolate dehydrogenase: implications for the catalytic mechanism of aldehyde dehydrogenases. *Biochemistry*.

[7] Cobessi D., Tête-Favier F., Marchal S., Branlant G., Aubry A. (2000). Structural and biochemical investigations of the catalytic mechanism of an NADP-dependent aldehyde dehydrogenase from *Streptococcus mutans*. *Journal of Molecular Biology*.

[8] González-Segura L., Rudiño-Piñera E., Muñoz-Clares R. A., Horjales E. (2009). The crystal structure of a ternary complex of betaine aldehyde dehydrogenase from *Pseudomonas aeruginosa* provides new insight into the reaction mechanism and shows a novel binding mode of the 2′-phosphate of NADP+ and a novel cation binding site. *Journal of Molecular Biology*.

[9] Ahvazi B., Coulombe R., Delarge M. (2000). Crystal structure of the NADP^+^-dependent aldehyde dehydrogenase from *Vibrio harveyi*: structural implications for cofactor specificity and affinity. *Biochemical Journal*.

[10] Tsybovsky Y., Krupenko S. A. (2011). Conserved catalytic residues of the ALDH1L1 aldehyde dehydrogenase domain control binding and discharging of the coenzyme. *Journal of Biological Chemistry*.

[11] D'Ambrosio K., Pailot A., Talfournier F. (2006). The first crystal structure of a thioacylenzyme intermediate in the ALDH family: new coenzyme conformation and relevance to catalysis. *Biochemistry*.

[12] Marchal S., Rahuel-Clermont S., Branlant G. (2000). Role of glutamate-268 in the catalytic mechanism of nonphosphorylating glyceraldehyde-3-phosphate dehydrogenase from *Streptococcus mutans*. *Biochemistry*.

[13] Vedadi M., Szittner R., Smillie L., Meighen E. (1995). Involvement of cysteine 289 in the catalytic activity of an NADP^+^-specific fatty aldehyde dehydrogenase from *Vibrio harveyi*. *Biochemistry*.

[14] Weiner H. (1995). Involvement of glutamate 268 in the active site of human liver mitochondrial (class 2) aldehyde dehydrogenase as probed by site-directed mutagenesis. *Biochemistry®*.

[15] Muñoz-Clares R. A., González-Segura L., Riveros-Rosas H., Julián-Sánchez A. (2015). Amino acid residues that affect the basicity of the catalytic glutamate of the hydrolytic aldehyde dehydrogenases. *Chemico-Biological Interactions*.

[16] Perez-Miller S. J., Hurley T. D. (2003). Coenzyme isomerization is integral to catalysis in aldehyde dehydrogenase. *Biochemistry*.

[17] Johansson K., El-Ahmad M., Ramaswamy S., Hjelmqvist L., Jörnvall H., Eklund H. (1998). Structure of betaine aldehyde dehydrogenase at 2.1 Å resolution. *Protein Science*.

[18] Langendorf C. G., Key T. L. G., Fenalti G. (2010). The X-ray crystal structure of Escherichia coli succinic semialdehyde dehydrogenase; structural insights into NADP+/enzyme interactions. *PLoS ONE*.

[19] Di Costanzo L., Gomez G. A., Christianson D. W. (2007). Crystal structure of lactaldehyde dehydrogenase from *Escherichia coli* and inferences regarding substrate and cofactor specificity. *Journal of Molecular Biology*.

[20] Bains J., Boulanger M. J. (2008). Structural and biochemical characterization of a novel aldehyde dehydrogenase encoded by the benzoate oxidation pathway in *Burkholderia xenovorans* LB400. *Journal of Molecular Biology*.

[21] Díaz-Sánchez Á. G., González-Segura L., Rudiño-Piñera E., Lira-Rocha A., Torres-Larios A., Muñoz-Clares R. A. (2011). Novel NADPH-cysteine covalent adduct found in the active site of an aldehyde dehydrogenase. *Biochemical Journal*.

[22] Muñoz-Clares R. A., Díaz-Sánchez Á. G., González-Segura L., Montiel C. (2010). Kinetic and structural features of betaine aldehyde dehydrogenases: mechanistic and regulatory implications. *Archives of Biochemistry and Biophysics*.

[23] Tsybovsky Y., Malakhau Y., Strickland K. C., Krupenko S. A. (2013). The mechanism of discrimination between oxidized and reduced coenzyme in the aldehyde dehydrogenase domain of Aldh1l1. *Chemico-Biological Interactions*.

[24] Wymore T., Deerfield D. W., Hempel J. (2007). Mechanistic implications of the cysteine-nicotinamide adduct in aldehyde dehydrogenase based on quantum mechanical/molecular mechanical simulations. *Biochemistry*.

[25] Mardanov A. V., Gumerov V. M., Slobodkina G. B. (2012). Complete genome sequence of strain 1860, a crenarchaeon of the genus *Pyrobaculum* able to grow with various electron acceptors. *Journal of Bacteriology*.

[26] Pace C. N., Vajdos F., Fee L., Grimsley G., Gray T. (1995). How to measure and predict the molar absorption coefficient of a protein. *Protein Science*.

[27] Tanaka H., Inaka K., Sugiyama S. (2004). A simplified counter diffusion method combined with a 1D simulation program for optimizing crystallization conditions. *Journal of Synchrotron Radiation*.

[28] Takahashi S., Tsurumura T., Aritake K. (2010). High-quality crystals of human haematopoietic prostaglandin D synthase with novel inhibitors. *Acta Crystallographica Section F: Structural Biology and Crystallization Communications*.

[29] Kabsch W. (2010). XDS. Integration, scaling, space-group assignment and post-refinement. *Acta Crystallographica Section D: Biological Crystallography*.

[30] Otwinowski Z., Minor W. (1997). Processing of X-ray diffraction data collected in oscillation mode. *Methods in Enzymology*.

[31] Vagin A., Teplyakov A. (2010). Molecular replacement with MOLREP. *Acta Crystallographica Section F Structural Biology Communications*.

[32] Afonine P. V., Grosse-Kunstleve R. W., Echols N. (2012). Towards automated crystallographic structure refinement with phenix.refine. *Acta Crystallographica Section D: Biological Crystallography*.

[33] Emsley P., Lohkamp B., Scott W. G., Cowtan K. (2010). Features and development of Coot. *Acta Crystallographica Section D: Biological Crystallography*.

[34] Krupenko S. A., Wagner C., Cook R. J. (1997). Expression, purification, and properties of the aldehyde dehydrogenase homologous carboxyl-terminal domain of rat 10-formyltetrahydrofolate dehydrogenase. *Journal of Biological Chemistry*.

[35] Marchai S., Branlant G. (1999). Evidence for the chemical activation of essential Cys-302 upon cofactor binding to nonphosphorylating glyceraldehyde 3-phosphate dehydrogenase from *Streptococcus mutans*. *Biochemistry*.

[36] Lakowicz J. R. (2006). *Principles of Fluorescence Spectroscopy*.

[37] Takahashi K., Weiner H., Hu J. H. J. (1980). Increase in the stoichiometry of the functioning active sites of horse liver aldehyde dehydrogenase in the presence of magnesium ions. *Archives of Biochemistry and Biophysics*.

[38] Keller M. A., Zander U., Fuchs J. E. (2014). A gatekeeper helix determines the substrate specificity of Sjögren-Larsson Syndrome enzyme fatty aldehyde dehydrogenase. *Nature Communications*.

[39] Steinmetz C. G., Xie P., Weiner H., Hurley T. D. (1997). Structure of mitochondrial aldehyde dehydrogenase: the genetic component of ethanol aversion. *Structure*.

[40] Huo L., Davis I., Liu F. (2015). Crystallographic and spectroscopic snapshots reveal a dehydrogenase in action. *Nature Communications*.

