# Supplementary material for: NADP-Dependent Aldehyde Dehydrogenase from Archaeon Pyrobaculum sp.1860: Structural and Functional Features
Source: Archaea. 2016 Nov 10;2016:9127857. doi: 10.1155/2016/9127857 (PMC5121451; doi:10.1155/2016/9127857)
Supplement: Supplementary file 1 — Supplementary material contains (1) description of the determination of Kd values using Scatchard analysis; (2) Figures S1, S2 and S3(a,b) illustrating changes in the emission spectra of apo form of AlDHPyr1147 upon titration with coenzyme at 60°C; (3) Table S1 that lists conformations of the catalytic residues and the coenzyme in the models of AlDHPyr1147; (4) Figure 4S illustrating Nucleotide binding site structure of the binary AlDHPyr1147-NADP+ complex. [file 9127857.f1.docx]

**Supplementary**

**Determination of Kd values**

The Kd values were determined using Scatchard analysis applied for binding of ligand molecules of one type to macromolecule with identical independent binding sites. The dissociation constant for n equivalent sites is defined by the equation Kd = (n×E_t_-EL)(L_t_-EL)/EL, where L_t_ and E_t_ are the total concentrations of the ligand and of the enzyme with n binding sites, EL is the concentration of ligand-site complex. EL is obtained from equation EL = α×E_t_×n, where, α is the occupancy of the enzyme binding sites, calculated from α = (F_max_ - F)/(F_max_-F_min_), where F is observed fluorescence signal, F_max_ is maximal signal (no bound NADP), F_min_ is minimal signal (all binding sites are saturated with NADP). Plotting α/(L_t_ – α×n×E_t_) against (1-α) with n = 4 (4×E_t_ is concentration of monomers with one identical binding site), a straight line with slope 1/Kd is obtained. Polynomial approximation α (L_t_) defined by equation L_t_ = α×n×E_t_ + α/(1-α)×Kd gives Kd and n. Data were analyzed using the software Origin 8.0.

**Figure S1.** Changes in the tryptophan fluorescence spectrum of apo form of AlDHPyr1147 upon titration with solution of NADP (black lines) and solution of NADPH (red lines) at 60°C. Excitation is at 297 nm. Solid lines – normalized spectra of 3.6 μM of apo form of AlDHPyr1147 (subunit concentration), dash lines – normalized spectra after addition of 15 μM NADP and 15 μM NADPH, respectively.

**Figure S2.** Changes in the emission spectrum of apo form of AlDHPyr1147 upon titration with solution of NADP+ at 60°C. Excitation wavelength is 297 nm, [AlDHPyr1147] = 3.6 μM (subunit concentration), [NADP+] = 0 - 15.2 μM, 50 mM sodium pyrophosphate buffer, pH 8.8, 100 mM NaCl.

**Figure S3a**. Changes in the emission spectrum of apo form of AlDHPyr1147 upon titration with solution of NADPH at 60°C. Excitation wavelength is 297 nm, [AlDHPyr1147] = 1.36 μM (subunit concentration), [NADPH]=0 – 9.0μM, 50 mM sodium pyrophosphate buffer, pH 8.8, 100 mM NaCl.

**Figure S3b**. Changes in the emission spectrum of apo form of AlDHPyr1147 upon titration with solution of NADPH at 60°C. Excitation wavelength is 330 nm, [AlDHPyr1147] = 1.36 μM (subunit concentration), [NADPH] = 0 – 9.0μM, 50 mM sodium pyrophosphate buffer, pH 8.8, 100 mM NaCl. AlDHPyr1147 + NADPH was incubated for 20 min at 60°C before each measurement. Black lines - emission spectra of NADPH, 0, 1 μM, 2 μM, 3 μM, 5 μM, 7 μM, 9 μM. Red lines - emission spectra of AlDHPyr1147 + NADPH. Insert: dependence of [F(AlDHPyr1147 + NADPH ) – F(NADPH)] upon NADPH concentration.

Table S1: Conformations of the catalytic residues and the coenzyme in the models of AlDHPyr1147.

| Models of AlDHPyr1147 | Crystallization conditions | Conformations of mobile elements in the structures | |
| --- | --- | --- | --- |
|  |  | Coenzyme | Catalytic residues |
| Apo | Crystallization of the apo form of AlDHPyr1147 | No | Cys287: the side chain is directed towards the coenzyme-binding pocket.  Glu253: the side chain is directed towards the coenzyme-binding pocket (“**inside**” conformation). |
| Holo-1 | Crystallization of AlDHPyr1147 as isolated | NADP(H) is in the “out” and “hydride transfer” conformations in all subunits except B; in B, NADP(H) is only in the “hydride transfer” conformation; the nicotinamide ring is disordered in all subunits | Cys287 in two conformations:  1) the side chain is directed towards the coenzyme-binding pocket;  2) the side chain is directed away from the coenzyme-binding pocket  Glu253: the side chain is directed towards the coenzyme-binding pocket (“**inside**” conformation); partially disordered. |
| Holo-2 | Cocrystallization of the apo-form of AlDHPyr1147 with NADP | NADP+ is in the “out” and “hydride transfer” conformations in all subunits, the nicotinamide ring is disordered in all subunits | Cys287 in two conformations:  1) the side chain is directed towards the coenzyme-binding pocket;  2) the side chain is directed away from the coenzyme-binding pocket.  Glu253: in two conformations:  1) the side chain is directed towards the coenzyme-binding pocket (“**inside**” conformation);  2) the side chain is directed away from the coenzyme-binding pocket (“**intermediate**” conformation). |
| Holo-3 | Crystal of the apo form is soaked in NADP and isobutyral solution | NADP+ is in the “hydride transfer” conformation; occupancy for the nicotinamide ring is 70 - 80 % | Cys287 in two conformations:  1) the side chain is directed towards the coenzyme-binding pocket in 30 % of subunits;  2) the side chain is directed away from the coenzyme-binding pocket in 70% of subunits.  Glu253: the side chain is directed away from the coenzyme-binding pocket (“**intermediate**” conformation); partially disordered. |
| Ternary complex | Cocrystallization of the apo form with NADP+ and isobutyraldehyde | NADP+ in the “hydride transfer” conformation, fully ordered. | Cys287: the side chain is directed away from the coenzyme-binding pocket.  Glu253: the side chain is directed away from the coenzyme-binding pocket (“**intermediate**” conformation). |

**Figure S4**. Nucleotide binding site structure of the binary AlDHPyr1147-NADP+ complex. Hydrogen bonds are shown by dashed lines.
